# The impact of analgesic on EMG and other biosignals in a postoperative setting

**DOI:** 10.3389/fmed.2023.1038154

**Published:** 2023-03-15

**Authors:** Sascha Gruss, Matthias Schmid, Steffen Walter, Benedikt Schick, Lena Holler, Eberhard Barth

**Affiliations:** ^1^Department of Psychosomatic Medicine and Psychotherapy, University Hospital of Ulm, Ulm, Germany; ^2^Department of Anaesthesiology and Intensive Care Medicine, University Hospital of Ulm, Ulm, Germany

**Keywords:** biosignals, morphine equivalents, pain medication, surrogate markers, features, automatic pain recognition

## Abstract

**Background:**

In the clinical context, the assessment of pain in patients with inadequate communication skills is standardly performed externally by trained medical staff. Automated pain recognition (APR) could make a significant contribution here. Hereby, pain responses are captured using mainly video cams and biosignal sensors. Primary, the automated monitoring of pain during the onset of analgesic sedation has the highest relevance in intensive care medicine. In this context, facial electromyography (EMG) represents an alternative to recording facial expressions *via* video in terms of data security. In the present study, specific physiological signals were analyzed to determine, whether a distinction can be made between pre-and post-analgesic administration in a postoperative setting. Explicitly, the significance of the facial EMG regarding the operationalization of the effect of analgesia was tested.

**Methods:**

*N* = 38 patients scheduled for surgical intervention where prospectively recruited. After the procedure the patients were transferred to intermediate care. Biosignals were recorded and all doses of analgesic sedations were carefully documented until they were transferred back to the general ward.

**Results:**

Almost every biosignal feature is able to distinguish significantly between ‘*before*’ and ‘*after*’ pain medication. We found the highest effect sizes (*r* = 0.56) for the facial EMG.

**Conclusion:**

The results of the present study, findings from research based on the BioVid and X-ITE pain datasets, staff and patient acceptance indicate that it would now be appropriate to develop an APR prototype.

## 1. Introduction

In the clinical context, the assessment of pain in patients with inadequate communication skills is still standardly performed externally by trained medical staff. This is usually done with external observation scales and is limited to and valid only at the time of assessment. Fine-grained continuous documentation is not possible given the lack of personnel time. Essentially, observational scales rely on a classification of pain components (1) that are used to assign pain-associated stress. Such indirect pain indicators are also referred to as surrogate markers. Mainly, surrogate markers such as facial expressions, vocalization, posture, and respiration are numerically assessed to determine a total pain score. Especially, the behavioral pain scale (BPS) is used in intensive care units when patients are unresponsive (2). All external pain assessment observation scales are only meaningful to a limited extent. In addition, a subjective coating by the observer is unconsciously included in the assessment. Automated pain recognition (APR) could make a significant contribution here. It is a visionary means to exploit valid and robust pain response patterns that can be measured multimodally (= multiple signals) for a dynamic, high temporal resolution, objective automated pain recognition system. The idea and development of an APR was primarily driven by the awareness that patients with impaired communication skills are at high risk of over-or underuse of pain-relieving analgesics. In recent years, machine learning algorithms related to APR have continuously emerged and evolved with the intention of further exploring pain configuration. The validation of these algorithms was always performed on healthy volunteers and pain patients. In this respect, APR actually represents an external objective observation method in which artificial intelligence is combined with hardware and software components with the goal of robustly and validly detecting pain. Werner et al. (3) and Frisch et al. (4) provide an excellent overview of the development status of the APR methodology for this purpose. The current focus of APR is on the detection of pain and its distinction between the classes “no pain” vs. “pain threshold” vs. “pain tolerance.” In this context, pain responses are explicitly recorded using video (for facial expressions (5, 6) and gestures (7–9)), microphones (for paralinguistics (10, 11)), and biosignal sensors which capture physiological activity, such as electrodermal activity [EDA], skin temperature, muscle activity [= electromyography, EMG], cardiac activity [= electrocardiogram, ECG] (12–16). The first commercial prototypes used only a single sensor for a rudimentary pain detection (17). In contrast, Medasense^1^ designed a device that records EDA, heart rate variability, and temperature synchronously for use during surgical procedures. There are preliminary empirical findings on postoperative use. Several studies showed that pain recognition rates were significantly higher (18) when different modalities were synchronously merged (19) than rather using a single signal/modality.

Explicitly, checking pain intensity during the onset of analgesic sedation is of utmost relevance in intensive care medicine to avoid over-or underdosing. Therefore, an automated monitoring of pain could be of highest relevance. The basics for such a method have not been sufficiently investigated so far.

Nevertheless, the recording of facial expressions, gestures, and paralinguistics is critical for ethical reasons and data security. In this context, EMGs of the facial muscles *zygomaticus major* and *corrugator supercilii* represent an alternative to recording facial expressions *via* video. They can serve as a substitute by appropriate interpretation of their activities. In general, very few studies have investigated the applications of facial EMGs (20, 21).

The following research questions are the focus of this brief report:

In the present study, the activities of specific physiological signals (facial EMG, ECG, skin conductance level (SCL) and Temperature (TEMP)) were analyzed to determine, whether a substantial distinction can be made between pre-and post-analgesic administration in a postoperative setting. Explicitly, the significance of the facial EMG in operationalization the effect of analgesia was investigated.

## 2. Methods

### 2.1. Patients

Patients scheduled for a surgical intervention within the next 3 days with the necessity of post-monitoring in an intermediate care (ICM) unit were prospectively enrolled in the study after completion of a written, informed consent. They had to be at least 18 years old, oriented and capable of providing information. Exclusion criteria were pregnancy, neurological diseases or having a pacemaker or defibrillator. All procedures were carried out in the ICM (35 beds) at the University Medical Center of Ulm, Germany. From September 29th, 2020 to April 13th, 2021, *N* = 38 (men = 28, women = 10) patients were included in the study. The ages ranged from 36 to 81 years, mean age was 62.3 years. Unfortunately, due to COVID-19 restrictions, the target sample size of 50 could not been reached.

### 2.2. Procedures and morphine equivalents

**Figure 1:**
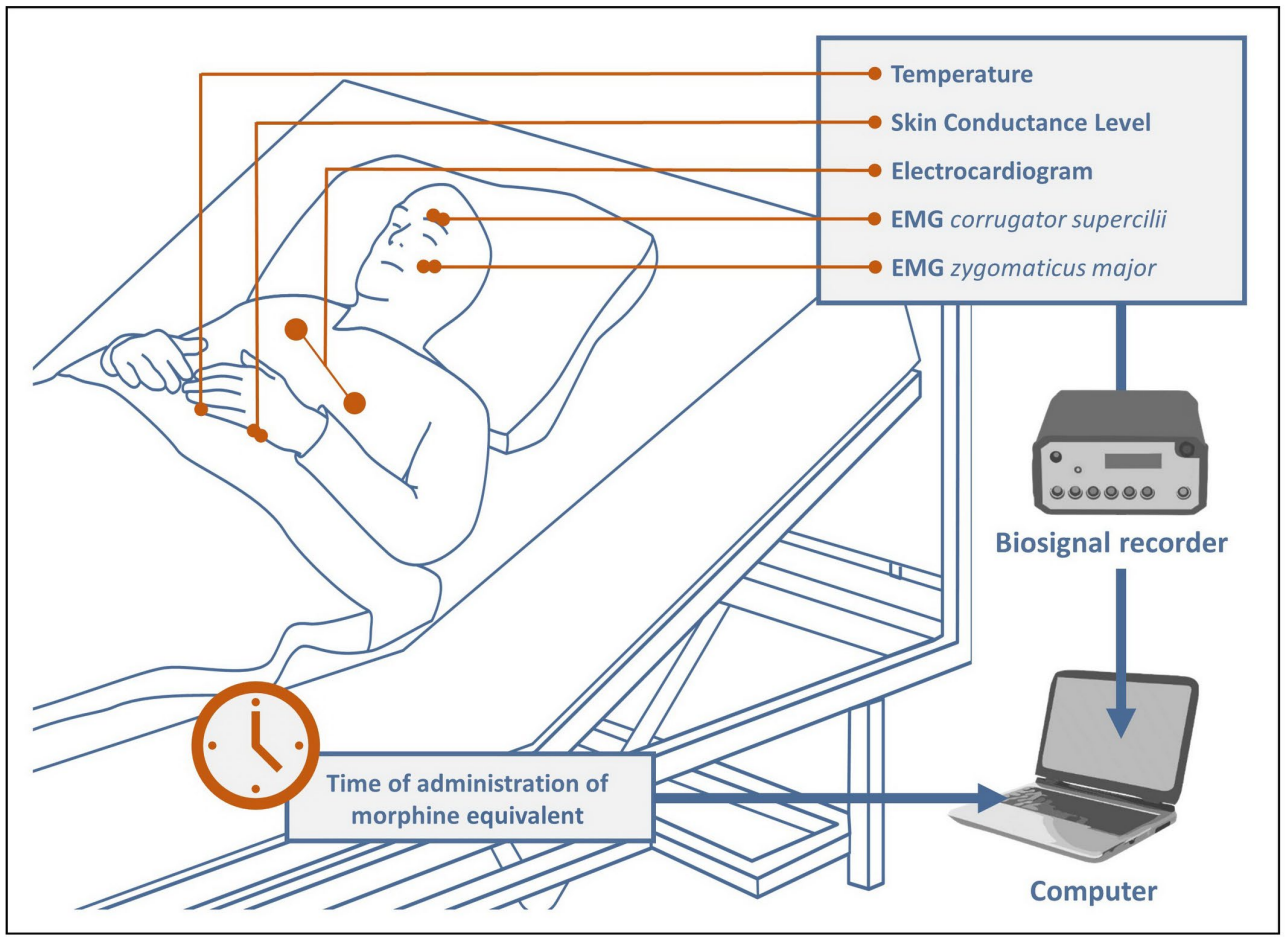
Study setup. EMG ≙ Electromyography.

After the surgical procedure, the patients were transferred to intermediate care. There, after attaching electrodes to them to acquire and monitor biosignals relevant for the study, recording was started immediately and continued continuously throughout the patient’s stay in the ICM (see Figure 1). All patients remained under constant observation by 2 medical doctoral candidates until they were able to be transferred back to the general ward. During this time, all pain medications were meticulously documented and manually marked in the recordings. The main analgesics administered were tramadol, tilidine, piritramide, oxycodone, and remifentanil. To make the different drugs comparable, a conversion to their morphine equivalents was performed based on (22, 23) as follows: 1 mg morphine ≅ 0.1 mg tramadol/tilidine ≅ 0.7 mg piritramide ≅ 2 mg oxycodone ≅ 100 mg remifentanil/fentanyl ≅ 1,000 mg sufentanil. The average time the patients spent in intermediate care was about 22.8 h, the average number of analgesic administrations was 10.4 with an average dose of 5.45 mg (converted to morphine equivalents).

### 2.3. Recorded biosignals

Physiological signals were recorded at a sample rate of 1,024 Hz, using the NeXus-10-amplifier and its associated software “BioTrace+ NX10” from Mind Media.^2^ All biosensors were applied according to the instructions in Gruss et al. (24). Bipolar pairs of Ag/AgCl electrodes were placed over the right *corrugator supercilii* and right *zygomaticus major* muscles to measure the Electromyography of the face. To record the ECG, three Ag/AgCl electrodes were attached to the patients’ upper body. Two more electrodes were attached on the bottom edge of the left hand to capture the SCL. At last, to obtain the peripheral body temperature, a temperature (TEMP) sensor was fixed to the tip of the left little finger with a medical tape. The software offered the possibility of manually setting markers during recording. Each time a pain medication was administered (see 2.2), a corresponding marker was set to mark the exact time in the biosignal recordings.

### 2.4. Biosignal processing and feature extraction

The processing of all physiological signals and subsequent feature extraction was done utilizing the “MATLAB 2018b” software from MathWorks.^3^ Firstly, all biosignals were downsampled to 512 Hz to speed up the processing procedures. In a next step, each type of biosignal was individually filtered as follows:

TEMP & ECG: We applied a moving average to both of the signals to smoothen their data. For the temperature we chose a 513 data-points and for the ECG a 67 data-points sliding window. In addition, all ECG signals were detrended.

SCL: The signal was filtered with a 20 Hz-low-pass filter and also smoothened with a 1,025 data-points sliding window.

EMG: The 2 EMG channels were processed with a 3rd-order Butterworth bandpass filter with cut-off frequencies of 20 and 250 Hz. Next, a Hilbert-transformation was performed and absolute values of the resulted signal were obtained. Finally, the data were low-pass filtered with a cut-off frequency of 4 Hz (25).

For statistical calculations, we extracted features for all biosignals from relevant data windows. The windows had a length of 60 s and were cut out of the signals directly ‘*before*’ and 5 min ‘*after*’ pain medication if possible. Those analgesic administrations had been manually marked in the biosignal recordings (see 2.3). All windows were visually inspected for artifacts and outliers. If necessary and doable, corrupted segments were corrected, otherwise the data window was discarded. Finally, features were derived that differed in number and type depending on the biosignal. With future machine learning in mind, we chose relevant features for biosignals suggested by Gruss et al. (16) and Werner et al. (3). To calculate the only ECG feature, we additionally used the Pan-Tompkins QRS-algorithm to detect R-peaks in the signal (26). They were grouped according to their corresponding windows (‘*before*’ and ‘*after*’ pain medication). All specific features are shown in Table 1.

**Table 1:** Extracted features for each biosignal.

Biosignal	Specific signal feature
Electromyography (EMG) *musculus corrugator supercilii & musculus zygomaticus major*	f1_EMG = maximum [in μV]
	f2_EMG = minimum [in μV]
	f3_EMG = root mean square [in μV]
	f4_EMG = mean of local maxima [in μV]
	f5_EMG = mean of local minima [in μV]
	f6_EMG = peak to peak of the means of local extrema [in μV]
	f7_EMG = mean [in μV]
	f8_EMG = standard deviation [in μV]
	f9_EMG = difference between max and min [in μV]
	f10_EMG = interquartile range Q3-Q1
Skin Conductance Level (SCL)	f1_SCL = maximum [in μS]
	f2_SCL = peak to peak of the means of local extrema [in μS]
	f3_SCL = slope of linear regression line [in μS/Hz]
Temperature (TEMP)	f1_TEMP = maximum [in °C]
	f2_TEMP = slope of linear regression line [in °C/Hz]
Electrocardiogram (ECG)	f1_ECG = mean length of successive RR intervals [in ms]

μV ≙ microvolt, μS ≙ microsiemens, °C ≙ degrees Celsius, Hz ≙ hertz, ms ≙ millisecond, fx_y ≙ feature number x of biosignal y.

### 2.5. Statistical Analysis

For all statistical tests we used the software “SPSS Statistics 28”.^4^ We performed non-parametric comparisons between the groups ‘*before*’ and ‘*after*’ for all biosignals and their associated features. With regard to the repetition of measurements, a Wilcoxon signed-rank test for dependent samples was chosen. A *p*-value of <0.05 indicated statistical significance. In addition, for each *p*-value, we calculatedthe associated effect size *r* using the formula *r* =$\;\left|\frac{Z}{\sqrt{N}}\right|$, *Z* = teststatistic, *N* = number of samples. Effect size measures help to assess the practical relevance of the results of statistical tests. Cohen’s classification (27) was used to assess the size of the effect: 0.1 ≤ *r* < 0.3 = small, 0.3 ≤ *r* < 0.5 = medium, and *r* ≥ 0.5 = large effect. Additionally, we were interested in whether the activities of both facial muscles were related to each other. In this sense, we calculated the differences of the related ‘*before*’ and ‘*after*’ values for all features for both muscles if both EMG signals were usable within the same window. The differences represented the change in activity and were used for non-parametric correlation analyses of the corrugator and the associated zygomaticus activities. Spearman’s ρ (= rho) and associated *p*-values were used to assess the association: 0.1 ≤ |ρ| < 0.3 = small, 0.3 ≤ |ρ| < 0.5 = moderate, and |ρ| ≥ 0.5 = strong correlation (28).

## 3. Results

After discarding unusable data windows, at least more than 330 samples per biosignal were left to examine statistically significant differences (*N*_EMG_corrugator_ = 349, *N*_EMG_zygomaticus_ = 344, *N*_SCL_ = 331, *N*_TEMP_ = 350, *N*_ECG_ = 349). A sample always consisted of a ‘*before*’ event and its related ‘*after*’ event. For the correlation analyses, there were a total of 310 samples, since both facial EMGs within the same sample always had to be qualitatively usable. Table 2 shows the results of the Wilcoxon tests for the comparisons of ‘*before*’ and ‘*after*’ groups for each biosignal feature.

**Table 2:** Biosignal activity represented through features directly ‘*before*’ and 5 min ‘*after*’ pain medication: results of Wilcoxon signed-rank tests and associated effect sizes.

Biosignal	Signal feature	Before pain medication		After pain medication		*p*	*r*	*Z*	Change (*before* – *after*)
		mean	±SD	mean	±SD				
EMG *corrugator supercilii* (*N* = 349)	f1_EMG	52.52	38.39	32.74	35.47	0.000	0.47	−8.78	↘
	f2_EMG	2.47	2.69	2.74	2.73	0.191	0.07	−1.31	↗
	f3_EMG	13.48	9.39	8.92	6.96	0.000	0.51	−9.46	↘
	f4_EMG	12.30	8.65	8.49	6.54	0.000	0.48	−9.05	↘
	f5_EMG	8.77	6.36	6.48	4.73	0.000	0.41	−7.70	↘
	f6_EMG	3.52	2.75	2.02	2.46	0.000	0.56	−10.54	↘
	f7_EMG	10.87	7.65	7.58	5.59	0.000	0.48	−8.91	↘
	f8_EMG	7.29	6.33	3.89	4.92	0.000	0.52	−9.73	↘
	f9_EMG	50.05	38.63	30.00	35.85	0.000	0.47	−8.82	↘
	f10_EMG	5.79	6.41	2.88	4.27	0.000	0.52	−9.73	↘
EMG *zygomaticus* *major* (*N* = 344)	f1_EMG	46.11	42.10	27.15	36.37	0.000	0.52	−9.64	↘
	f2_EMG	2.46	2.19	2.75	2.26	0.867	0.01	−0.17	↗
	f3_EMG	11.16	7.24	7.87	6.50	0.000	0.49	−9.08	↘
	f4_EMG	10.18	6.19	7.52	5.77	0.000	0.46	−8.53	↘
	f5_EMG	7.27	4.55	5.82	4.13	0.000	0.39	−7.25	↘
	f6_EMG	2.91	2.23	1.70	2.21	0.000	0.53	−9.82	↘
	f7_EMG	8.97	5.40	6.78	4.95	0.000	0.45	−8.38	↘
	f8_EMG	6.14	5.45	3.25	4.83	0.000	0.55	−10.26	↘
	f9_EMG	43.66	42.57	24.40	36.30	0.000	0.52	−9.72	↘
	f10_EMG	4.89	5.34	2.48	3.78	0.000	0.53	−9.82	↘
SCL (*N* = 331)	f1_SCL	2.30	0.91	2.14	0.63	0.000	0.31	−5.70	↘
	f2_SCL	0.41	0.49	0.48	0.68	0.000	0.30	−5.53	↗
	f3_SCL	1.59E-06	7.77E-05	0.00	0.00	0.019	0.13	−2.34	↗
TEMP (*N* = 350)	f1_TEMP	34.99	3.31	35.11	3.37	0.004	0.15	−2.87	↗
	f2_TEMP	−1.17E-05	2.81E-05	0.00	0.00	0.000	0.42	−7.85	↗
ECG (*N* = 349)	f1_ECG	1395.58	213.69	1443.53	234.19	0.000	0.51	−9.44	↗

EMG ≙ Electromyography, SCL ≙ Skin Conductance Level, TEMP ≙ Temperature, ECG ≙ Electrocardiogram, sd ≙ standard deviation, N ≙ sample size, fx_y ≙ feature number x of biosignal y, *p* ≙ significance (2-tailed), size, *r* ≙ effect size, *Z* ≙ test statistic.

Almost every feature is able to distinguish significantly between the groups. The *p*-values hereby range from 0.019 to 0.000 with medium (*r* ≥ 0.3) to large (*r* ≥ 0.5) effect sizes. The largest effect size within this study was found for feature “f6_EMG” of EMG_*corrugator_supercilii* with *r* = 0.56 (*p* = 0.000). Features “f3_SCL” (*p* = 0.019, *r* = 0.13) and “f1_TEMP” (*p* = 0.004, *r* = 0.15) show high significant differences but have small effect sizes. No differences between the groups were found for features “f2_EMG” of EMG_*corrugator_supercilii* (*p* = 0.191) and “f2_EMG” of EMG_*zygomaticus_major* (*p* = 0.867). Except for the two features mentioned above, all biosignal activity changed to the expected directions. Muscle and SCL activity turned down, peripheral body temperature slightly increased and heart beat slowed down. All findings strongly indicate a reduced sympathicus activity after pain medication.

Regarding a related activity of the two facial muscles, Table 3 shows that almost all correlation coefficients reveal highly significant moderate to strong correlations, ranging from ρ = 0.383** to 0.634**. Only “diff_f2_C/Z” shows a weak correlation (ρ = 0.158**). All in all, the activities of the two facial muscles do not seem to be independent of each other.

**Table 3:** Results of the non-parametric correlation analyses between EMG_corrugator_supercilii and EMG_zygomaticus_major.

EMG *corrugator* *supercilii* (*N* = 310)	EMG *zygomaticus* *major* (*N* = 310)	Correlation coefficient spearman’s rho *ρ*	*p*
diff_f1_C	diff_f1_Z	0.485**	<0.001
diff_f2_C	diff_f2_Z	0.158**	0.005
diff_f3_C	diff_f3_Z	0.489**	<0.001
diff_f4_C	diff_f4_Z	0.461**	<0.001
diff_f5_C	diff_f5_Z	0.383**	<0.001
diff_f6_C	diff_f6_Z	0.634**	<0.001
diff_f7_C	diff_f7_Z	0.444**	<0.001
diff_f8_C	diff_f8_Z	0.515**	<0.001
diff_f9_C	diff_f9_Z	0.489**	<0.001
diff_f10_C	diff_f10_Z	0.463**	<0.001

EMG ≙ Electromyography, N ≙ sample size, diff_x_C/Z ≙ difference(s) of feature number x of biosignal C/Z (C = corrugator, Z = zygomaticus), *p* ≙ significance (2-tailed). Each diff_fx was calculated as “diff_fx” = “fx_*after* pain medication” – “fx_*before* pain medication.”

## 4. Discussion

The fact that analgesia can be directly observed and assessed in a postoperative setting, e.g., with the BPS, is part of the S3-guideline “Analgesia, Sedation and Delirium management in Intensive Care.” That pain intensity has an influence on the autonomic nervous system has been shown in numerous studies (see introduction). However, it has not been operationalized to what extent the activities of biosignals are influenced after an analgesic administration. In this brief report the operationalization of an analgesic effect was proven statistically significant for the recorded biosignals EMG, SCL, ECG, and TEMP. This is an indication that physiological signals are suitable to check for an analgesic effect and should be transferred into an AI-based prototype. This would be a key benefit for clinical staff.

Walter et al. (29) showed that the majority of clinical staff in the ICU would prefer a biosignal-based pain detection. In relation to this, facial EMG would have the benefit of replacing computer vision, in terms of cost effectiveness and data security. For clinical staff, the use of computer vision systems would be more complex, more demanding, and ultimately more error-prone as a result.

Despite initial skepticism of the authors regarding the (patient) acceptability of the facial EMG sensors, the continuous measurement of facial EMG was shown to be unproblematic.

Limitations:

1. The sample is relatively small due to the complexity and duration of measurements and Covid-19 pandemic restrictions.
2. The extent to which a particular analgesic dosage affected the activities of the various biosignals was not investigated.
3. Why feature “f2_EMG” (= minimum of EMG signal) does not show a significant difference, cannot be verified at the moment.

Outlook:

1. Further studies should statistically examine the influence of a certain dosage of analgesics on the sympathetic/parasympathetic response.
2. Findings from research based on the BioVid (30) & X-ITE (24) datasets, the results of the present study, staff acceptance (29), and patient acceptance indicate that it would now be appropriate to develop and test an APR prototype.

## Data availability statement

The datasets presented in this article are not readily available because of data security. Requests to access the datasets should be directed to sascha.gruss@uni-ulm.de.

## Ethics statement

The studies involving human participants were reviewed and approved by Ethics committee of the Ulm University (No. 441/19). The patients/participants provided their written informed consent to participate in this study.

## Author contributions

SG: study design, literature search, data analysis, and writing. BS: critical revision and writing. MS and LH: data collection. EB: study design, critical revision, and data interpretation. SW: study design, literature search, and writing. All authors contributed to the article and approved the submitted version.

## Conflict of interest

The authors declare that the research was conducted in the absence of any commercial or financial relationships that could be construed as a potential conflict of interest.

## Publisher’s note

All claims expressed in this article are solely those of the authors and do not necessarily represent those of their affiliated organizations, or those of the publisher, the editors and the reviewers. Any product that may be evaluated in this article, or claim that may be made by its manufacturer, is not guaranteed or endorsed by the publisher.

## Footnotes

^1^ www.medasense.com

^2^ www.mindmedia.com

^3^ www.mathworks.com

^4^ www.ibm.com/products/spss-statistics

## Abbreviations

Ag/Cl: silver chloride
APR: Automated Pain Recognition
COVID-19: Coronavirus Disease 2019
EMG: Electromyography
ECG: Electrocardiogram
Hz: hertz
ICM: Intermediate Care
SCL: Skin Conductance Level
TEMP: Temperature
